# Systemic Organizational Changes in Health Care and Their Implications for Job Crafting in Nursing: A Systematic Review

**DOI:** 10.1155/jonm/9204110

**Published:** 2026-07-29

**Authors:** Jolanta Lubaś, Jadwiga Klukow, Joanna Czerwik-Marcinkowska, Sylwia Skorupska, Szymon Zmorzyński

**Affiliations:** ^1^ Department of Nursing, Institute of Health, State University of Applied Sciences in Krosno, Rynek Street 1, 34-400, Krosno, Poland, pwsz.konin.edu.pl; ^2^ Chair of Nursing Management and Clinical Nursing, Institute of Clinical Sciences, Faculty of Health Sciences, Academy of Zamosc, Pereca Street 2, 22-400, Zamosc, Poland; ^3^ Institute of Biology, Jan Kochanowski University, Stefana Żeromskiego Street 5, 25-369, Kielce, Poland, ujk.edu.pl; ^4^ Institute of Human Sciences, Faculty of Health Sciences, Academy of Zamosc, Pereca Street 2, 22-400, Zamosc, Poland

## Abstract

**Background:**

Shaping the work environment in nursing reflects the concept of job crafting, which involves proactive strategies by nurses to redefine tasks, social interactions, and cognitive framing of work, aiming to increase its perceived significance and job satisfaction. The systematic review aims to comprehensively synthesize all relevant research evidence on job crafting within the framework of the healthcare system.

**Methodology:**

A review was conducted, using a systematic search of selected literature related to job crafting in the healthcare system, with a particular focus on nurses. A systematic search was conducted in PubMed/MEDLINE, Scopus, and CINAHL. The search included studies published between January 2015 and January 2025. The prepared review used the Preferred Reporting Items for Systematic Reviews and Meta‐Analyses (PRISMA) checklist. A predefined search strategy was systematically applied to the identified records. The work was registered in the PROSPERO database.

**Results:**

A total of twenty‐five full‐text records were included in the final review. Based on the analysis of key domains, such as work engagement (including work efficiency), commitment to work, job satisfaction, intention to remain in the profession, quality of care, and burnout, directions for the development of job crafting were identified. A review of studies indicates that job crafting is positively and significantly associated with work engagement, commitment to work, and job satisfaction (*r* = 0.22–0.70) and negatively associated with burnout (*r* = −0.41 to −0.24). Although individual analyses have reported a lack of statistical significance or different directions of dependence, the overall picture confirms the beneficial role of job crafting for employee well‐being. Additionally, the analysis highlighted the need for further research in these areas. While numerous studies have examined the concept of job crafting in nursing, the review underscores a persistent gap in the literature, specifically concerning the linkage between employee motivation and proactive modifications of the work environment.

**Conclusions:**

Job crafting can help to reduce burnout among nurses by enabling them to adapt their tasks, relationships, and perceptions of work. Higher levels of job crafting are associated with greater work engagement, job satisfaction, and intention to remain in the profession, particularly when it involves modifying tasks and relationships. Job crafting is facilitated by autonomy, a supportive organizational culture, and supervisory support but hindered by rigid procedures, heavy workloads, staff shortages, and limited decision‐making opportunities. Although nurses’ knowledge, experience, and interpersonal skills are keys to effective job crafting, organizational systems often fail to utilize these competencies.

## 1. Introduction

Healthcare system professionals possess a set of skills, values, and knowledge, collectively referred to as core competencies, that enable them to perform their responsibilities effectively and in accordance with professional standards. These competencies constitute the fundamental requirements for practice within a given discipline and serve as a standard for assessing professional competence and development [1]. Nurses make up the largest percentage of workers in the healthcare system worldwide and play an important role in clinical treatment, disease prevention (preventive measures), and health promotion. This professional group plays a key role in providing appropriate care to patients, performing their work in an ever‐changing and demanding environment. Moreover, nurses often experience high stress in their daily patient care; so, fostering a supportive work environment and encouraging job crafting is crucial [2].

Shaping the work environment is a broader term than “job crafting” and refers to the process of shaping or influencing the work environment. Job crafting refers to the proactive modifications that employees make to the tasks they perform, how they perceive their work, and the social interactions they engage in. These modifications include task crafting (changing the type, scope, or sequencing of duties), cognitive crafting (changing the meaning of work), and relational crafting (adjusting social interactions at work) [3]. In nursing, job crafting enables nurses to actively shape their work environment. It refers to a proactive and creative process through which nurses modify their tasks, interpersonal interactions, or cognitive perceptions of work to enhance its personal relevance and meaning. This approach enables healthcare professionals to align their roles with individual strengths, interests, and values, thereby promoting occupational well‐being and mitigating the risk of professional burnout [4]. This involves reorganizing tasks to improve patient care, enhancing collaboration with colleagues, and reframing routine duties in a more meaningful way. Ultimately, this supports professional well‐being and reduces burnout [2]. Job crafting is proactive adaptation of one’s work by an employee in order to better align it with their skills, values, and preferences. However, due to high professional demands, limited resources, work‐family conflicts, and complex clinical work environments, nurses have been observed to experience symptoms of burnout, such as emotional exhaustion, cynicism, and/or reduced professional effectiveness [5, 6]. These phenomena negatively affect the quality of care, patient safety and satisfaction, productivity, and turnover rate, as well as physical and mental health of nurses [7, 8]. According to a nationwide survey conducted in the United States, 16.6%–30.0% of nurses (out of 3,957,661 samples) reported experiencing burnout and 31.5% of them reported burnout as a factor contributing to their decision to leave their jobs [9]. A retrospective study conducted in 12 European countries found that symptoms of burnout were present in 10%–78% of nurses [10]. In addition to providing comprehensive patient care, nurses are required to provide organizational management and maintain appropriate professional relationships in a rapidly changing and stressful environment. Factors, such as heavy workloads, staffing shortages, emotional exhaustion, and a perceived lack of support, contribute to job burnout and dissatisfaction, which can ultimately lead to a high intent to leave the workforce [4, 11]. To ensure proper patient care, nurses are expected to continuously improve their professional practice to meet demands based on available resources, which involves shaping their tasks, dealing with workplace stressors, and prioritizing tasks that are consistent with patient‐centered care [12]. Although burnout among nurses is well documented, there is still a limited understanding of the proactive strategies nurses can use themselves to mitigate these effects in their daily work.

The Job Demands–Resources (JD‐R) model developed by Demerouti et al. [13] has become a widely used framework for examining and addressing workplace characteristics. The model proposes two primary pathways: one linking job resources to motivational outcomes, such as work engagement, and another connecting high levels of job demands with strain. Job demands refer to the physical, social, or organizational aspects of work that require sustained effort and are associated with physiological and psychological costs [13]. Conversely, job resources are aspects of the job that facilitate the achievement of work goals, mitigate the impact of job demands and associated costs, and promote personal growth and development [13]. The JD‐R model suggests that high levels of job demands (e.g., heavy workloads or emotional demands) can lead to stress and burnout. In contrast, job resources (e.g., autonomy or social support) can promote engagement and well‐being [14, 15]. Within the JD‐R model, job crafting can be seen as a mechanism that allows nurses to adjust their tasks, interactions, and perceptions in order to better align their available resources with the demands placed on them. This mitigates burnout and enhances engagement [16]. This review focuses on studies that examine how nurses’ job crafting behaviors influence job demands, resources, burnout, and engagement, and is guided by the JD‐R model framework.

Healthcare systems worldwide are currently undergoing substantial organizational transformations, driven by demographic changes, workforce shortages, technological advancements, and the increasing demand for efficient, high‐quality care [17]. Reforms relating to staffing policies, cost containment strategies, the digitalization of healthcare services, and evolving models of patient‐centered care are having a significant impact on nurses’ work environments [18]. Such changes at the organizational and policy levels often modify job demands, professional responsibilities, and decision‐making autonomy, creating challenges and opportunities for nurses. In such contexts, job crafting may emerge as an adaptive strategy through which nurses can proactively adjust their tasks, relationships, and perceptions of work in response to evolving organizational conditions [19]. Therefore, it is essential to understand job crafting within the broader framework of healthcare system management in order to identify how systemic changes influence nurses’ ability to shape their work environment. With the development of healthcare systems, the role of nurses is evolving, as well as their job crafting in response to the increasing demands on this professional group.

Job crafting is defined as physical and cognitive changes made by individuals in the tasks or relational boundaries of their work, as well as self‐initiated changes that employees make to their own work requirements and resources to achieve and/or optimize their professional goals [3]. Lichtenthaler and Fischbach defined job crafting as modifications to specific job tasks and employees’ perceptions of them [20]. This concept is based on the employees’ reaction to organizational changes in the workplace [21]. The demanding nature of nursing work, coupled with increasing organizational pressures, makes nurses particularly vulnerable to occupational stress and burnout.

Although much research focuses on the personal resources, mental well‐being, and work engagement of individuals as determinants of job crafting, the influence of the broader organizational context of the healthcare system is analyzed far less frequently. However, changes in health policy, employment models, or management practices can significantly impact the opportunities available to nursing staff to modify their work. Therefore, it is crucial to take the organizational context into account when seeking to understand the role of job crafting in healthcare management. The aim of this systematic review is therefore to examine how job crafting functions as a strategic response to professional burnout in nursing, enhancing well‐being, engagement, and retention, particularly within the broader context of organizational and systemic challenges in contemporary healthcare systems. While many studies have examined the effects of job crafting on nurses, few have synthesized findings from various healthcare settings or explored the systemic and organizational factors affecting job crafting in nursing. Moreover, most studies analyze the correlations between variables and few examine whether job crafting programs actually improve nurses’ well‐being. However, there is a lack of longitudinal and intervention studies that assess the effectiveness of job crafting programs in improving the quality of patient care, and in enhancing the functioning of nursing teams, in various healthcare system contexts. The review primarily includes studies conducted in Asian healthcare settings. There is smaller representation from Europe and the Middle East, which may affect how generalizable the findings are. The present work posed the following questions: (I) Which aspects of job crafting specifically influence burnout levels among nurses? (II) What is the connection between job crafting and work engagement, job satisfaction, and the desire to continue working as a nurse? (III) How do nurses respond to organizational changes and stressful working conditions by adapting their job? (IV) What are the factors, both organizational and systemic, that either support or limit the potential for job crafting in the nursing environment? (V) To what extent can nurses’ competencies be used as a basis for them to modify their working conditions independently? To the authors’ knowledge, no comprehensive work exists that synthesizes research findings on job crafting in nursing, specifically addressing the aforementioned areas.

## 2. Survey Methodology

This review was conducted and reported in accordance with the Preferred Reporting Items for Systematic Reviews and Meta‐Analyses (PRISMA) checklist [22]. The search was limited to contemporary literature selected from leading journals focused on healthcare and system management, identified during a search of major electronic databases. The work was registered in the PROSPERO database with the number CRD420251070555. Studies published between January 2015 and January 2025 were eligible for inclusion.

### 2.1. Eligibility Criteria

The inclusion criteria covered original, full‐text, open‐access research articles published between January 2015 and January 2025. Eligible studies were written in English, focused specifically on the nursing profession, and employed the Job Crafting Scale as a research instrument.

Exclusion criteria included preprints, review articles, articles published in a language other than English, articles with insufficient information or limited access to the full text, and articles on professional groups other than nurses.

### 2.2. Information Sources

A review of PubMed/MEDLINE, Scopus, and CINAHL databases was conducted to identify content suitable for the literature review being prepared. These databases were selected as those that would be most relevant to the subject of the review and had the broadest range of relevant content.

### 2.3. Search Strategy

A series of text words, synonyms, and subject headings were developed for the main constructs. The search strategy employed operators to combine keywords and synonyms related to job crafting and the nursing profession. The core search query was structured as follows: (job crafting) AND (nurs∗ OR health care) AND (motivation OR engagement OR efficiency OR performance OR commitment OR satisfaction OR intent to stay OR burnout OR quality of care OR self‐efficacy). The search formula and wildcards were flexibly modified by combining subject terms and free words. The search included studies published between January 2015 and January 2025.

### 2.4. Selection Process

Two independent reviewers (J.L. and J.K.) conducted record selection based on titles and abstracts, followed by the full texts of the publications. Any discrepancies regarding the eligibility of a particular study for review were resolved through discussion or with a third reviewer (S.Z.). Studies that did not meet the predefined inclusion criteria were excluded from further analysis.

### 2.5. Data Collection Process

Data from each report were collected manually by two independent reviewers (J.L. and J.K.), using a previously developed data extraction form. This form was prepared at the planning stage of the review and included such elements as bibliographic data (authors, year of publication, country), purpose of the study, study design, sample characteristics (size, professional group, context), measurement tools used, key findings, and authors’ conclusions. Reviewers worked independently, and any discrepancies in the information collected were resolved through discussion or with a third reviewer (S.Z.).

### 2.6. Data Items

The systematic review defined the data set that was collected from each report for analysis. The Scopus database initially yielded 1043 articles, from which 370 publications were selected for further analysis. After applying the inclusion criteria, 10 articles qualified for final review from the Scopus database. The largest number of publications was identified in 2024 (*n* = 136). A PubMed/MEDLINE database initial search yielded 255 results, of which 31 publications were retrieved after restriction to open‐access articles. The distribution of the number of publications by year was as follows: 2018—1 article, 2020—2, 2021—3, 2022—5, 2023—8, 2024—11, 2025—1. After deduplication and evaluation of titles and abstracts, 12 articles were included in the review (from PubMed/MEDLINE). A total of 472 records were retrieved from the CINAHL database. Of these, 142 publications were selected for full‐text evaluation. After applying the inclusion criteria, three articles remained and were incorporated into the final review.

### 2.7. Study Risk‐of‐Bias Assessment

The assessment of the bias risk of each study was made independently by two reviewers based on predetermined methodological criteria. Factors, such as clarity of the study’s purpose, appropriateness of the research design to the questions posed, method of sampling, quality and relevance of the measurement tools used, clarity of results presentation, and presence of potential conflicts of interest, were considered. In the case of discrepancies between assessments, decisions were made through discussion or with a third reviewer. The risk‐of‐bias assessment served as one of the criteria in the final interpretation of the quality and reliability of the collected data.

### 2.8. Effect of Measures

The included studies primarily reported associations between job crafting and professional outcomes, employing correlation coefficients (Pearson’s *r* or Spearman’s rho), regression coefficients, and mediation or moderation analyses. The most frequently examined outcomes included work engagement, job satisfaction, burnout, job embeddedness, commitment to work, and characteristics of the nursing practice environment (Table 1). As the included studies differed substantially in terms of their study design, outcome measures, statistical approaches, and reported metrics, it was deemed inappropriate to conduct a quantitative meta‐analysis. Therefore, the findings were synthesized narratively.

**TABLE 1:** Overview of trends for job crafting in nursing within the included literature.

	Feature	References	The described relationship	Key conclusions
Job crafting and	Work engagement	[2, 23–26]	Statistically significant positive correlations (*r* ranges from 0.25 to 0.42). In the study of [23], the statistically insignificant results were observed in the case of the second survey (T2). Insignificant results were observed by [2].	Job crafting enhances individual work engagement and supports nurses’ well‐being. However, collaborative job crafting at the ward level shows no clear link to positive or negative outcomes. Its effectiveness is strengthened in a healthy nursing practice environment.
	Commitment to work	[27]	Statistically significant positive correlation (*r* = 0.69)	Through active job crafting, nurses can significantly increase their commitment to their work.
	Job satisfaction	[28, 29]	Statistically significant positive correlations (*r* ranges from 0.22 to 0.70)	Job crafting, particularly when carried out collaboratively, mediates the relationship between leader‐member exchange and nurses’ job and life satisfaction. This impact is strengthened by factors, such as job security, fair compensation, meaningful work, and positive relationships with supervisors.
	Job embeddedness	[23, 30–32]	Statistically significant negative correlation (*r* = −0.54)^∗^ ^∗^The study by Yun et al. does not provide the value of this correlation.	Job crafting has been shown to make nurses feel more fulfilled in their jobs and less detached from their work, although it can be counterproductive if there is too much conflict in their roles.
	Burnout	[33–35]	Statistically significant negative correlations (*r* ranges from −0.41 to −0.24)	Job crafting is a key strategy in demanding clinical environments, supporting nurses’ well‐being by buffering stress and burnout, enabling skill development and confidence building, and fostering stronger interpersonal relationships.
	Nursing practice environment	[36–39]	Statistically significant positive correlation (*r* = 0.50)	The perception of the staff appraisal system is an element of the organizational environment and nursing practice. Improving the nursing practice environment is essential for promoting person‐centered care. Educational programs integrating clinical storytelling and case‐based learning can enhance nurses’ critical reflection skills and job crafting, fostering an environment that nurtures professional development and efficient patient care.
	Character strengths	[36, 37]	Statistically significant positive correlation (*r* = 0.70)	The results emphasize the importance of nurturing nurses’ character strengths, such as empathy, resilience, and interpersonal skills. Enhancing these personal attributes alongside critical reflection and job crafting enables nurses to deliver person‐centered care and respond adaptively to the demands of clinical practice.

### 2.9. Synthesis Methods

To qualify studies for synthesis, a selection was made according to defined inclusion and exclusion criteria. For each synthesis, only studies with measurable results linked to variables of interest, such as job burnout, commitment to work, job satisfaction, or intention to stay in the profession, were included. The qualification process was conducted by two independent reviewers based on an analysis of the full texts, and decisions were made jointly in case of discrepancies.

### 2.10. Reporting Bias Assessment

The systematic review used a qualitative assessment of the risk of bias due to missing data in the syntheses, caused by incomplete reporting of results, among other things. For each included study, it was analyzed whether all relevant outcomes consistent with the stated objectives of the study were reported, and whether it was possible to clearly identify effects for the variables analyzed (e.g., burnout). Statistical tools were not used, as no meta‐analysis was conducted. The potential risk of bias associated with incomplete reporting was addressed in the narrative interpretation (Table 2).

**TABLE 2:** Summary of the papers included in the literature review.

No.	Author	Type of publication	Country	Number of cases	Questionnaires used	Risk of bias in studies	Conclusions related to job crafting
1.	Hwang and Shin [37]	A cross‐sectional study	Korea	132	‐ The Job Crafting Scale ‐ The critical reflection competency ‐ The nursing practice environment ‐ The Person‐Centered Care Scale	‐ Respondents are selected employees and may not represent all staff. ‐ The majority of participants in this study had fewer than 10 years of professional experience. ‐ All variables were assessed based on nurses’ self‐reports, which may introduce subjectivity and potential cognitive errors. ‐ The sample was relatively small.	The findings indicated that critical reflection competency and job crafting were key determinants of person‐centered care among nurses working in tertiary general hospitals.

2.	Zhang et al. [40]	A cross‐sectional study	China	655	‐ The Job Crafting Scale ‐ The Copenhagen Psychosocial Questionnaire ‐ The Workplace Well‐Being Scale	‐ Single data collection time point. ‐ The long‐term effects of job crafting on nurses’ well‐being have not been studied. ‐ Pregnant nurses and those with a history of mental illness were excluded. This may have influenced the discrepancy in results—because these groups may have had different experiences of well‐being or job crafting.	Job crafting was identified as a key factor supporting work well‐being by buffering the negative effects of poor health and enabling proactive coping with work‐related stress. Promoting job crafting behaviors may serve as an effective strategy to enhance nurses’ well‐being in demanding clinical environments.

3.	Saleh et al. [27]	Research article	Saudi Arabia	381	‐ The Job Crafting Scale ‐ Appreciative Leadership Scale ‐ Workplace Belongingness Scale ‐ Affective Organizational Commitment Scale	‐ The sample was relatively younger than the average age of nurses in the country. ‐ Participants in the study came from two urban university hospitals. ‐ All variables were assessed based on nurses’ self‐reports, which may introduce subjectivity and potential cognitive errors.	By actively engaging in job crafting, nurses can significantly improve their commitment to work.

4.	Zhang et al. [35]	Research article	China	714	‐ The Job Crafting Scale ‐ Perceived supervisor autonomy support scale ‐ Occupational Well‐Being Scale ‐ Career Shocks Scale	‐ The design, involving cross‐sectional and single‐source data collection, is inadequate for determining causality. ‐ All variables were assessed based on nurses’ self‐reports, which may introduce subjectivity and potential cognitive errors. ‐ The study utilizes structural equation modeling to test hypotheses, and any violations of its assumptions could introduce bias into the results.	‐ The positive career shocks can enhance nurses’ occupational well‐being by promoting job crafting. ‐ Implementing job crafting interventions may help nurses find greater meaning in their work and improve their well‐being.

5.	Han [2]	Research article	Korea	207	‐ The Job Crafting Scale ‐ The Utrecht Work Engagement Scale ‐ The Well‐being Measurement Tool	‐ The design, involving cross‐sectional and single‐source data collection, is inadequate for determining causality. ‐ All variables were assessed based on nurses’ self‐reports, which may introduce subjectivity and potential cognitive errors. ‐ The sample was relatively small.	Job crafting enhances work engagement, which then improves well‐being.

6.	Li et al. [41]	Research article	China	1006	‐ The Job Crafting Scale ‐ The Inventory of Character Strengths	‐ Nurses in this study were recruited from only four tertiary hospitals, where differences in nursing environments and job crafting practices may limit the generalizability of the findings to other tertiary hospitals in China. ‐ All variables were assessed based on nurses’ self‐reports, which may introduce subjectivity and potential cognitive errors.	The character strengths explained over half of the variance in job crafting, highlighting their role as a key determinant of nurses’ job crafting behavior.

7.	Guo et al. [33]	Research article	China	1235	‐ The Job Crafting Scale ‐ The Maslach Burnout Inventory‐General Survey ‐ The Leisure Crafting Scale	‐ The design, involving cross‐sectional and single‐source data collection, is inadequate for determining causality. ‐ All variables were assessed based on nurses’ self‐reports, which may introduce subjectivity and potential cognitive errors. ‐ Nurses on sick leave or in the process of leaving their jobs were excluded from the study, which may have led to an underestimation of obtained results.	Despite experiencing symptoms of burnout, nurses tend to engage in job crafting to enhance their clinical skills, boost confidence, strengthen interpersonal relationships, and support personal development.

8.	Roczniewska and Bakker [34]	Research article	Poland	81	‐ The Job Crafting Scale ‐ The general survey comprised demographic questions and trait‐level measures.	‐ All variables were assessed based on nurses’ self‐reports, which may introduce subjectivity and potential cognitive errors. ‐ The observed relationships may be subject to error due to the use of the same measurement method (known as common methodological variance). ‐ The sample was relatively small.	Job crafting was identified as a key predictor of burnout, highlighting its critical role in mitigating burnout symptoms.

9.	Ghazzawi et al. [42]	Research article	Lebanon	547	‐ The Job Crafting Scale ‐ Subscale of Autonomy at Work Scale ‐ The Positive and Negative Affect Schedule ‐ The Satisfaction with Life Scale ‐ The Self‐report Scale (to measure creativity)	‐ The presence of self‐selection bias. Specifically, nurses who are overworked or nearing burnout may have been less likely to participate due to limited time, energy, or motivation. This could result in an underrepresentation of individuals experiencing the most severe symptoms of burnout, thereby skewing the findings and potentially underestimating the true extent of burnout and its impact on job crafting behaviors within the nursing population.	Job crafting among nurses positively affects their well‐being, with benefits extending beyond work. Creativity helps nurses adapt their work behaviors through job crafting. Personality traits, such as agreeableness, conscientiousness, honesty, and emotional stability, influence engagement in job crafting.

10.	Baig et al. [43]	Research article	Pakistan	283	‐ The Job Crafting Scale ‐ The Diversity Climate Scale ‐ The Innovative Work Behavior Scale	‐ Single data collection time point. ‐ The design, involving cross‐sectional and single‐source data collection, is inadequate for determining causality. ‐ Did not examine any moderator. Such a role of leader (leadership style) as a moderator between the relationship of job crafting and innovative work behavior can be assessed. ‐ The sample was relatively small.	Employees who feel comfortable and optimistic about diversity are more likely to engage in job crafting, which in turn fosters innovation.

11.	Pan et al. [28]	Research article	Taiwan	263	‐ The Job Crafting Scale ‐ Job Satisfaction Scale	‐ Conducting the survey in just one province limits the applicability of the results to nurses in other hospitals. ‐ Data were collected from individuals who were easiest to find and asked to participate in the survey. ‐ The sample was relatively small.	The relationship between leader‐member exchange and job and life satisfaction is mediated in part by individual and collaborative job crafting, with collaborative shaping playing a greater role.

12.	Zhang et al. [26]	Research article	China	350	‐ The Job Crafting Scale ‐ The Work Engagement Scale	‐ All variables were assessed based on nurses’ self‐reports, which may introduce subjectivity and potential cognitive errors. ‐ The design, involving cross‐sectional and single‐source data collection, is inadequate for determining causality. ‐ The sample was relatively small.	An important factor in favor of job crafting is job involvement.

13.	Chang et al. [44]	Research article	Korea	220	‐ The Job Crafting Scale ‐ The Korean Happiness Index ‐ The Practice Environment ‐ Scale of the Nursing Work Index	‐ Data were collected from individuals who were easiest to find and asked to participate in the survey. ‐ The design, involving cross‐sectional data collection, is inadequate for determining causality. ‐ The sample was relatively small.	The individual‐level happiness, rather than organizational‐level nursing practice environments, was significantly associated with job crafting.

14.	Iida et al. [23]	Prospective cohort study	Japan	2478	‐ The Job Crafting Scale ‐ The Utrecht Work Engagement Scale ‐ The WHO Health and Work Performance Questionnaire ‐ The K6 questionnaire ‐ The Intention to Leave Scale ‐ The Brief Job Stress Questionnaire ‐ The Effort‐Reward Imbalance (ERI) Questionnaire	‐ The number of participants and wards included in the analysis represented only a small fraction of the expected sample size. ‐ All variables were assessed based on nurses’ self‐reports, which may introduce subjectivity and potential cognitive errors. ‐ The independent and dependent variables were measured concurrently, without any temporal separation.	The collaborative job crafting at the ward level was not significantly associated with either positive outcomes (such as work engagement and performance) or negative outcomes (such as psychological distress and intention to leave).

15.	Sidin et al. [29]	Research article	Indonesia	155	‐ The Job Crafting Scale ‐ Job Satisfaction Scale	‐ The design, involving cross‐sectional data collection, is inadequate for determining causality. ‐ All variables were assessed based on nurses’ self‐reports, which may introduce subjectivity and potential cognitive errors. ‐ The sample was relatively small.	Job crafting may contribute to increased job satisfaction, particularly when supported by additional factors, such as job security, adequate compensation, meaningful work, and positive relationships with immediate supervisors.

16.	Yun et al. [31]	Research article	Korea	260	‐ The Job Crafting Scale ‐ The Role Conflict Scale ‐ The Positive Psychological Capital Questionnaire ‐ The Social Support Instrument ‐ Job Embeddedness Tool	‐ The design, involving cross‐sectional data collection, is inadequate for determining causality. ‐ Conducting the survey in general (and general tertiary) hospitals limits the applicability of the results to nurses in other hospitals. ‐ The sample was relatively small.	Job crafting positively influences job embeddedness, particularly when individuals possess high levels of psychological capital and receive strong social support. However, job crafting may also lead to increased role conflict, and when this conflict becomes substantial, it can hinder job crafting efforts and ultimately reduce job embeddedness.

17.	Rodríguez‐García et al. [11]	A cross‐sectional study	Spain	284	‐ The Job Crafting Scale	‐ Data were collected from individuals who were easiest to find and asked to participate in the survey. ‐ All variables were assessed based on nurses’ self‐reports, which may introduce subjectivity and potential cognitive errors. ‐ The sample was relatively small.	The development of job crafting competencies aimed at reducing hindering job demands may be supported by interventions including workload and time management training, supportive leadership, access to adequate resources, the promotion of autonomy, team‐based collaboration, and mental health initiatives.

18.	Yeo and Ha [45]	Research article	Korea	127	‐ The Job Crafting Scale ‐ The Korean Occupational Stress Scale‐Short Form ‐ The Psychological Capital Questionnaire ‐ The Person–Job Fit Scale	‐ Data were collected from individuals who were easiest to find and asked to participate in the survey. ‐ Conducting the survey in select blood centers limits the applicability of the results to nurses in other hospitals/centers. ‐ The sample was relatively small.	Job crafting emerges as a key component in understanding how nurses shape their work to enhance job satisfaction.

19.	Kato et al. [24]	Research article	Japan	309	‐ The Job Crafting Scale ‐ The Utrecht Work Engagement Scale ‐ The Practice Environment Scale of the Nursing Work Index ‐ Strength‐oriented Care Attitudes Scale ‐ The 5‐item World Health Organization Well‐Being Index	‐ Conducting the survey in select three psychiatric hospitals limits the applicability of the results to nurses in other hospitals. ‐ Data were collected from individuals who were easiest to find and asked to participate in the survey. ‐ The design, involving cross‐sectional, is inadequate for determining causality. ‐ The sample was relatively small.	Job crafting and a healthy nursing practice environment can enhance work engagement.

20.	Topa and Aranda‐Carmena [25]	Research article	Spain	699 (study 1) 498 (study 2) 308 (study 3)	‐ The Job Crafting Scale ‐ The Utrecht Work Engagement Scale ‐ The Performance Scale (In‐Role Behaviors)	‐ All variables were assessed based on nurses’ self‐reports, which may introduce subjectivity and potential cognitive errors. ‐ The lack of comprehensive data regarding the type of hospital (public vs. private), which were only collected in study 2, limits the ability to draw conclusions about potential differences in job crafting behaviors among nurses employed in these two organizational settings. ‐ The sample was relatively small.	Different types of job crafting behaviors vary in effectiveness and suitability depending on the specific work environment.

21.	Alharthi et al. [46]	Research article	Saudi Arabia	441	‐ The Job Crafting Scale ‐ The Oxford Happiness Questionnaire	‐ Conducting the survey in one hospital limits the applicability of the results to nurses in other hospitals. ‐ The use of email and WhatsApp for survey distribution, which was not fully aligned with the intended data collection procedure. ‐ The sample was relatively small.	Job crafting is positively and significantly associated with nurses’ happiness.

22.	Zohourparvaz and Vagharseyyedin [32]	Research article	Iran	332	‐ The Job Crafting Scale ‐ The Global Measure of Job Embeddedness ‐ The Work Alienation Measure	‐ All variables were assessed based on nurses’ self‐reports, which may introduce subjectivity and potential cognitive errors. ‐ The design, involving cross‐sectional, is inadequate for determining causality. ‐ The sample was relatively small.	Approximately half of the variance in nurses’ work alienation can be explained by their levels of job crafting and job embeddedness.

23.	Khalil et al. [38]	Research article	Egypt	105	‐ The Job Crafting Scale ‐ The Performance Appraisal System Questionnaire	‐ All variables were assessed based on nurses’ self‐reports, which may introduce subjectivity and potential cognitive errors. ‐ It is not known whether those who declined to participate differed systematically (e.g., whether they were more burnt out or less committed). ‐ The sample was relatively small.	A positive perception of the performance appraisal system was significantly associated with higher job crafting among nurses, with the result that those who held a positive perception of the system were more likely to engage in job crafting than those who held a negative perception.

24.	Ebrahimi and Fathi [30]	Research article	Iran	269	‐ The Job Crafting Scale ‐ Survey of Perceived Organizational Support ‐ Job Embeddedness Measure	‐ All variables were assessed based on nurses’ self‐reports, which may introduce subjectivity and potential cognitive errors. ‐ The article is missing a “Limitations” section.	Job embeddedness may influence the relationship between perceived organizational support and job crafting by acting as a contextual factor, suggesting that employees who are more embedded in their organization may be more likely to translate perceived support into proactive job redesign behaviors.

25.	Roczniewska and Puchalska‐Kamińska [39]	Research article	Poland	Study 1—267 Study 2—262	‐ The Job Crafting Scale ‐ Perceived Autonomy Scale	‐ The design, involving cross‐sectional data collection, is inadequate for determining causality. ‐ There is a possibility of confounding factors, such as proactive personality, influencing both job crafting and managerial status. Online data collection reduces control over responses and may lead to careless or duplicate responses.	Higher autonomy, particularly among managers, is associated with more frequent job crafting, which involves increasing job resources and challenging demands. Managers engage in job crafting more often than lower level employees, which highlights the importance of organizational rank. The autonomy associated with managerial roles enables active job redesign, whereas newer managers are more likely to reduce hindering job demands as a form of job crafting.

## 3. Results

**FIGURE 1:**
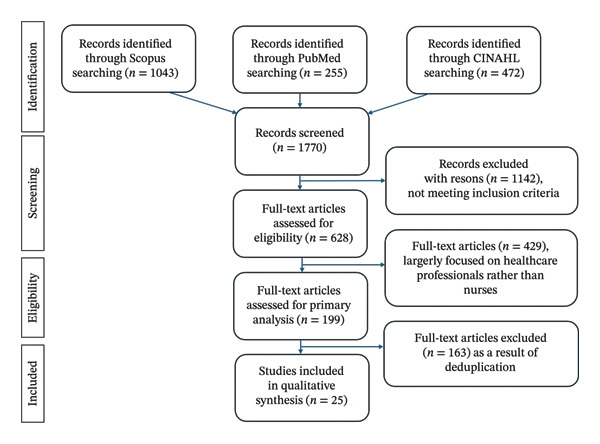
The PRISMA flowchart.

The review yielded a total of 1770 relevant literature items. After reviewing titles and abstracts, 628 papers were included. After deduplication, a total of 25 literature items were obtained that met the selection criteria. The PRISMA flowchart is shown in Figure 1. In Table 2, a summary of the data obtained during the literature search is presented, including the questionnaires used.

### 3.1. General Characteristics of Included Studies

The included studies were synthesized according to the primary outcomes investigated. Five major domains emerged from the analysis: work engagement; commitment to work; job satisfaction; job embeddedness; burnout; and nursing practice environment.

**FIGURE 2:**
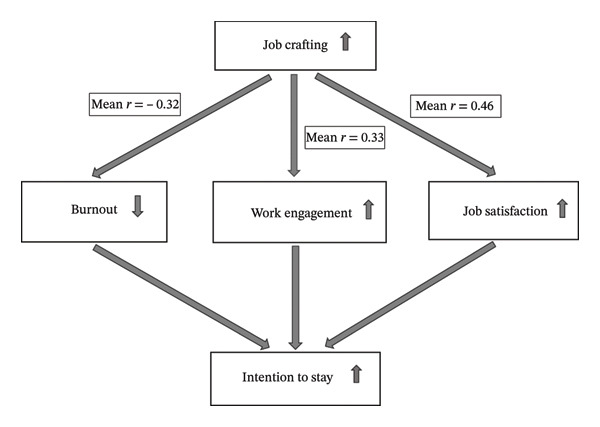
Conceptual relationships between job crafting and key nursing outcomes. Job crafting enables nurses to proactively modify their tasks, their perception of work, and their social interactions. Evidence from the included studies suggests that job crafting is linked to lower burnout levels and higher work engagement and job satisfaction. This, in turn, may encourage nurses to stay in the profession.

All studies included in the analysis were quantitative. Six studies were from the cross‐sectional study type [11, 30, 37–40]. One study was a prospective cohort study type [23] (Table 2). Several areas related to job crafting around the world were identified, as well as some gaps regarding this phenomenon in the professional group of nurses. The interrelationship between job crafting and areas related to management in the healthcare system has been used in several works [26, 33, 41]. Recent studies have shown that job crafting is positively related to the degree of happiness of nurses [44], well‐being [15, 47, 48], personal commitment, maintaining motivation, and quality of care [49]. However, most studies have focused on the relationship between job crafting and work engagement [2, 23–26, 50] (Figure 2).

### 3.2. Detailed Characteristics of Included Studies

Included studies are listed in Table 2. Job crafting represents a creative aspect of nursing practice, whereby nurses adapt tasks to better meet patients’ needs, ultimately enhancing patient well‐being. In the process of reshaping their roles, nurses may encounter conflicts with established job expectations. Critical reflection competency and job crafting were found to influence person‐centered care [36, 37].

#### 3.2.1. Work Engagement

Work engagement was the most frequently investigated outcome. Five studies reported a positive correlation between job crafting and work engagement, with coefficients ranging from 0.25 to 0.42 [2, 23–26] (Table 1). Job crafting led to emotional well‐being and positively affected work engagement [2]. In longitudinal studies, this effect does not always persist over time—for example, in the cohort study by Iida et al. [23], no long‐term relationship was found between team job crafting and a sustained increase in engagement, suggesting the need to consider environmental and organizational factors [23]. These findings suggest that collaborative job crafting may not consistently promote sustained engagement. As existing research has largely focused on its benefits, future studies should also consider potential drawbacks. Still, task crafting aimed at team development at the ward level may contribute to increased individual engagement, highlighting the potential role of nursing managers in fostering such environments [23]. These insights offer practical implications for the development of team‐based job crafting interventions, particularly those emphasizing collective growth as a means to enhance nurses’ engagement at work. Work engagement promotes nurses’ professional effectiveness, and job crafting plays a mediating role. Their increased engagement translates into better performance. The effectiveness of specific job crafting behaviors depends on their type. Not all forms are equally effective and appropriate in a nursing context. The results confirm that commitment affects job performance, and job crafting mediates this relationship [25]. Qualitative analysis showed that nurses engage in cognitive, relational, and task crafting to adapt their roles. Given the limited research in this area, further quantitative and qualitative studies are needed [45]. Enhancing work engagement is essential for improving outcomes, such as strength‐oriented care attitudes, mental health, and turnover intention, with job crafting, especially cognitive crafting, playing a key role in this process. Incorporating job crafting into clinical practice may be supported by improving the nursing practice environment through increased supervisor support, fostering harmonious relationships with physicians, and strengthening the foundations for quality care. Providing educational opportunities that encourage nurses to reflect on the value and meaning of their work can serve as an effective strategy to promote cognitive crafting [24].

Many studies analyze correlations. However, there is a lack of in‐depth analysis of the underlying mechanisms. For example, research on nurses suggests that job crafting influences work engagement via various mediating factors, including positive psychological capital, the work environment, and job resources [24, 25]. However, the reasons why and the conditions under which job crafting increases engagement are still not fully understood. Job crafting can take various forms, such as task, relational, and cognitive crafting. Research suggests that these types do not all have the same impact on work performance [25]. Research is needed into the impact of specific forms of job crafting on nurses’ engagement and job satisfaction, and how this differs. While many articles focus primarily on individual factors, the influence of management style, workload, and team structure is examined less frequently [28, 51]. It is well known, however, that the work environment influences both job crafting and work engagement. Most studies are cross‐sectional and based on questionnaires. There is a lack of longitudinal studies, which would enable us to establish whether the relationship between job crafting and work engagement is causal.

#### 3.2.2. Commitment to Work and Job Embeddedness

Several studies have demonstrated a positive relationship between job crafting and organizational attachment, as indicated by work commitment, job embeddedness, intention to remain in the profession, and a sense of belonging in the workplace (Table 1). A strong positive correlation was observed between job crafting and work commitment (*r* = 0.69) [27] (Figure 2). Furthermore, lower levels of job crafting were associated with stronger intentions to leave both the current workplace and the nursing profession. Taken together, these findings suggest that job crafting may contribute to workforce retention by strengthening nurses’ attachment to their professional roles. Job crafting behaviors exert a significant positive impact on workplace belonging, underscoring their critical role in fostering a sense of inclusion among nurses. In addition, job crafting behaviors contribute positively to commitment, underscoring their significance in fostering stronger organizational attachment [27]. Furthermore, factors, such as job autonomy, a sense of meaningful work, and positive collegial relationships, can promote job embeddedness through the facilitation of job crafting [31]. Lower levels of the *Decreasing hindering job demands* dimension of job crafting among nurses appear to be associated with a stronger intention to leave both their current workplace and the nursing profession as a whole. This tendency also correlates with a reduced intention to remain in their current workplace. Given the limited body of research on job crafting in the nursing context, this observation represents a novel insight that warrants further investigation in future studies [11]. Using a cross‐sectional design, Ebrahimi and Fathi [30] reported a positive association between perceived organizational support and job crafting, with job embeddedness acting as a moderating variable [30]. However, as the study was correlational in nature, the findings do not allow for causal interpretations regarding the role of job crafting in enhancing organizational attachment or retention. Therefore, the observed relationships should be interpreted as concurrent associations rather than as evidence of directional effects. As the demands and complexity of their work grow, job crafting becomes essential for employees to effectively manage and adapt to these challenges.

#### 3.2.3. Burnout

Some studies indicate that job crafting can alleviate burnout symptoms by improving access to job resources and strengthening social support [33, 34] (Table 1). Nurses who actively engage in job crafting tend to report better perceived health and demonstrate a stronger capacity to manage stress and prevent burnout [40]. Job crafting was the most important predictor of burnout [33]. Despite experiencing some symptoms of burnout, nurses actively engage in job crafting to enhance their clinical skills, build confidence, strengthen interpersonal relationships, and support personal growth. Job crafting was identified as a key predictor of burnout, highlighting its critical role in mitigating burnout symptoms. Therefore, nurse managers should foster a supportive environment that encourages job crafting and implement strategies to enhance these behaviors, ultimately reducing burnout and improving both clinical performance and organizational commitment [34]. Nurses experiencing burnout have a reduced capacity to leverage peaks in self‐regulatory resources to enhance their performance through job crafting, highlighting the need for tailored interventions specifically designed for those facing high levels of burnout [34]. In hospital settings in China, Japan, and South Korea, job crafting primarily influences job satisfaction and meaningfulness. In contrast, European and American studies tend to observe its impact on team engagement and burnout reduction [2, 26, 41]. This suggests that the effects of job crafting may be partly conditioned by culture and organizational factors, with different patterns of influence emerging in various work environments and healthcare models.

#### 3.2.4. Job Satisfaction and Well‐Being

Job crafting was found to moderate the relationship between personal perceived health and work well‐being, as well as the mediated pathway involving the psychosocial work environment. This suggests that job crafting enables nurses to make adaptive adjustments in response to varying work conditions. When the psychosocial work environment impacts perceived health, job crafting may function as a personal strategy that supports adaptation and resilience. The study by Li et al. identified a moderate to strong positive correlation between character strengths and job crafting among nurses in tertiary hospitals in China (Table 2). Character strengths were found to significantly predict job crafting behaviors, explaining up to 81% of the variance. As one of the first studies to explore this relationship within the nursing profession, it highlights the potential of leveraging individual character strengths to promote proactive job redesign among nurses [41]. Extraversion showed no relationship with any dimension of job crafting. This finding may be unique to the nursing profession, where traits, such as assertiveness and dominance, are less advantageous due to the strong emphasis on adhering to regulations and effective teamwork [42].

The nurse managers and educators in the healthcare sector should foster a supportive work environment by involving nurses in decision‐making processes, promoting empowering leadership, and implementing supportive programs and initiatives aimed at enhancing both job happiness and engagement in job crafting [46]. Nurses who engage in job crafting tend to report higher job satisfaction, deliver more effective patient care, and contribute to the achievement of organizational goals. The individual level of happiness, rather than organizational factors, such as the nursing practice environment, is more strongly associated with job crafting among nurses. Administrative support, such as the provision of leisure programs, reduction of overtime hours, and promotion of work‐life balance, may enhance nurses’ well‐being and foster job crafting behaviors. Healthcare institutions should also identify key facilitators and barriers to job crafting across different roles and unit types, and offer tailored educational and training programs to support these efforts. Such organizational strategies may enhance overall employee well‐being and cultivate a work environment that promotes sustained engagement, particularly among nurses required to remain on duty for extended periods [46]. The healthcare organizations should promote a supportive diversity climate and encourage job crafting to stimulate employees’ creativity and innovation [43]. Both intrinsic and extrinsic factors play a significant role in shaping job crafting, and these same factors contribute to its association with job satisfaction [29]. Job crafting enables nurses to reshape or modify their work experience and environment to better cope with or adapt to career shocks, thereby enhancing their job satisfaction. For instance, success in a new role or a visible project can boost nurses’ confidence and motivate them to redesign their job tasks and social interactions at work, further increasing their occupational fulfillment [35]. It is essential for nurse managers to acknowledge these challenges by recognizing nurses’ perspectives and offering clear guidance regarding available options. Such support can foster motivation and effectiveness in performing professional duties. Leader‐member exchange constitutes a key predictor of both individual and collaborative forms of job crafting. This is consistent with the evidence that organizational rank and autonomy are associated with higher levels of job crafting, which is defined as the process of enhancing one’s job by making improvements to the way it is structured, performed, and experienced [39]. A collective approach contributes to strengthening job resources, which in turn helps to improve the employment outlook for individuals and communities. When nurses have access to sufficient resources within their hospitals, they are more likely to report higher job satisfaction and effectively cope with challenging situations [28]. Zhang et al. noted that although all nurses had the potential to engage in job crafting, not all of them actually demonstrated such behaviors in the workplace, revealing that the majority of nurses displayed a low level of job crafting [26]. Within this low job crafting group, efforts were more focused on reducing hindering work demands than on increasing job resources or seeking new challenges. Therefore, nursing managers should pay special attention to the group with low levels of job crafting and implement targeted measures to support the development of both job crafting and job commitment. To enhance the psychosocial work environment and foster higher levels of engagement, nursing managers should support nurses in recognizing the value of their work and adopting job crafting practices aligned with organizational objectives. As organizational performance appraisal systems have also been shown to be associated with job crafting behaviors, it is important to consider how these systems can be used to support and encourage these behaviors [38]. Key strategies include ensuring access to necessary job resources, involving nurses in decision‐making processes, and promoting participation in professional development initiatives [26]. To improve job satisfaction, it is recommended to develop employee assistance programs and educational initiatives that enhance person‐job fit, incorporate stress and emotional labor management strategies, and strengthen psychological capital [45]. Job crafting has the potential to enhance the overall sense of meaning nurses derive from their work, thereby improving job satisfaction and reducing feelings of work alienation. To support this process, healthcare organizations should promote job crafting by enabling nurses to modify aspects of their roles, offering professional development opportunities, managing workload demands, and fostering a work environment that strengthens collegial relationships [32]. Nurses with higher life satisfaction tended to demonstrate more proactive behaviors in shaping their job roles and responsibilities [44]. Strategies aimed at enhancing nurses’ happiness warrant comprehensive investigation. To promote adequate leisure time and improve overall well‐being, hospital organizations should consider measures, such as optimizing staffing levels, implementing flexible work schedules, and reducing overtime hours [44]. Research indicates that job crafting is strongly correlated with nurses’ engagement and job satisfaction. This, in turn, contributes to a better quality of care and professional well‐being [2, 23, 25]. In some cross‐sectional studies, these effects were more pronounced for job satisfaction than for burnout reduction, suggesting that job crafting may act more strongly as a motivational mechanism than as a protective factor against burnout [11, 40].

### 3.3. Emerging Trends and Research Areas

Job crafting involves changing the way one performs tasks, approaches to work, and relationships with others. What’s more, it also includes taking on more professional challenges, using available resources more efficiently, and learning to reduce work‐related burdens that interfere with one’s own needs [52]. A fresh paradigm of job crafting includes four dimensions, which are increasing organizational resources, raising job requirements, acquiring or increasing social resources, and reducing those requirements that hinder task execution. Nurses shape their professions by modifying the number of job requirements and available employment opportunities. Han observed a correlation between job crafting and work engagement and nurses’ psychological well‐being [2]. Psychological well‐being is based on an assessment of self‐acceptance, autonomy, and environmental, e.g., work and personal development [53]. In addition, the approach of job crafting allows supervisors and nurses to work together to reassess the goals, responsibilities, and interactive relationships in each position, which has a positive impact on employees [54]. The impact of leadership based on employee empowerment in shaping professional tasks has proven beneficial, as it leads to an increase in both structural and social resources and demands on nurses. Empowering leadership especially affects work engagement, increasing nurses’ cognitive skills [33].

Job crafting is adapted to suit the specific nature of the environment, and the forms it takes and the effects it has depend on the type of challenges nurses face in a given context. A review of the literature reveals that job crafting can manifest itself in various ways, depending on the area of specialization and the type of institution. In general hospitals, job crafting most often involves modifying tasks and reorganizing responsibilities. This enables nurses to manage their workload more effectively, thereby increasing their satisfaction and commitment to their work [26, 41]. In a psychiatric setting, cognitive and relational crafting—i.e., changes in how patients are interacted with and how difficult emotional situations are approached—are of greater importance. This improves the quality of psychological care and reduces professional burnout [44, 45]. In emergency departments and acute care settings, job crafting typically involves adapting quickly to changing requirements and time constraints. This approach fosters a sense of control over tasks and enhances the efficiency of teamwork [11, 26]. In clinical settings, nurses use task and relational crafting to innovate teaching methods and improve patient care. This increases engagement and professional development [2, 24]. To optimize benefits for staff and patients alike, job crafting strategies should be adapted to the unique characteristics of each ward, its specific patient population, and organizational requirements.

### 3.4. Relevant Conclusions for Healthcare Management

Job crafting can improve nurses’ well‐being and satisfaction, increasing their commitment to work and improving the quality of patient care [33, 35, 40]. A managerial approach grounded in empowerment fosters both individual and team‐oriented work, making resources more available and mitigating burnout [26, 28, 46]. Targeting groups with low levels of job crafting with interventions can strengthen engagement and a sense of meaning at work, particularly in environments where the tasks are demanding [11, 26]. The importance of individual resources, such as character traits, sense of happiness, and psychological capital, in job crafting demonstrates that personal and organizational factors are both crucial to optimizing the working environment for nurses [31, 41, 44]. Although some of the analyzed studies referred to the JD‐R model as the theoretical basis for job crafting, the model’s assumptions were not fully discussed in relation to the findings. In particular, the role of job demands and job resources in the nursing work environment, and the mechanisms through which job crafting may influence the balance between these factors, has not been analyzed simultaneously.

### 3.5. Competencies as a Basis for Job Crafting in Nursing

A growing body of literature suggests that nurses’ individual competencies and psychological resources are important factors in determining their engagement in job crafting and its effectiveness. Several studies have addressed the role of nurses’ competencies in engaging in job crafting behaviors, albeit indirectly. The ability to engage in critical reflection—that is, the capacity to analyze one’s own experiences and adjust one’s actions—was a strong predictor of person‐centered care in the study by Hwang and Shin [37]. This suggests that stronger self‐reflection skills can lead to a proactive transformation of work [37]. Nurses with stronger reflective skills may be better able to adapt their work tasks and interactions to meet the needs of their patients. Similarly, Li et al. [41] identified a strong correlation between character strengths and job crafting behaviors among nurses working in tertiary hospitals [41]. They found that character strengths significantly predicted job crafting, explaining a large proportion of the variance in proactive job redesign. Other studies suggest that psychological resources, such as psychological capital, perceived health, and overall life satisfaction, may facilitate job crafting behaviors by strengthening nurses’ ability to adapt to challenging work environments [31, 44]. Happiness and life satisfaction—that is, internal psychological resources—were significantly associated with higher levels of job crafting in the study by Chang et al. [44]. Overall, the reviewed literature suggests that individual competencies and personal resources could be important in enabling job crafting among nurses. However, the number of studies that explicitly examine competencies as a basis for job crafting is still small, suggesting that more research is needed in this area.

## 4. Discussion

This review focuses on various factors influencing the work environment among nurses, examining specific management areas within the healthcare system, including work productivity and efficiency, job satisfaction, intention to stay, quality of patient care, self‐efficacy, and occupational burnout. The growing number of challenges associated with job crafting underscores the global complexity of this phenomenon within the evolving context of healthcare systems. From the perspective of the JD‐R theory, job crafting refers to employees’ proactive behaviors aimed at reducing hindering job demands and enhancing job resources [55]. According to this model, high job demands lead to strain and burnout, while job resources encourage engagement and motivation at work. Job crafting is a proactive strategy that enables employees to adjust their job demands and resources. As job crafters, nurses appreciate the professional essence of nursing (cognitive crafting), leverage and refine the expertise gained through experience to enhance their practice, take on new responsibilities (task crafting), and foster close connections with patients and caregivers while engaging with colleagues through coaching and mentoring (relationship crafting) [3].

The working environment for nurses can vary depending on a number of factors, such as the tasks they perform and the specifics of their departments [56]. Within the nursing practice environment, critical reflection involves identifying and assessing problems by linking prior knowledge with past experiences, ultimately reframing the issue. Through this process, new perspectives emerge, leading to alternative solutions and adjustments in both understanding and behavior [57]. Consequently, job crafting by nurses is expected to shape the delivery of care in a way that aligns with patients’ interests and preferences. Nevertheless, studies exploring the link between job crafting and person‐centered care in tertiary hospitals are still scarce. Alongside the competencies of healthcare professionals, organizational and environmental conditions play a vital role as key enablers of person‐centered care [58]. Most studies indicate that nurses working in a hospital have low to medium levels of happiness, which may be related to the negative emotions they experience while caring for patients, as well as difficult working conditions and heavy workloads [46, 59]. High levels of happiness among employees are crucial, especially in nursing, where job tasks require providing care and/or appropriate therapy to others [60]. Patients require confidence, creative, caring, and committed caregivers, and each of these qualities is directly related to feelings of happiness. The work environment for nurses is demanding and can negatively impact the mental health of employees and their ability to provide quality care [60]. This may be related to the nature of the profession and professional skills, high awareness, and sound decision‐making necessary for success [60]. The strong correlation between job satisfaction and job crafting can be attributed to the combined influence of structural job resources and enhanced social resources on employees’ overall happiness.

Enhancing nurses’ professional competencies has been shown to foster higher workplace engagement, improve teamwork, and strengthen relationships among colleagues [21]. An increasing body of evidence indicates that, among nurses, high professional competence is linked to a strong work commitment [2, 50, 52]. Work engagement is regarded as a positive, fulfilling, and emotionally stable state in the workplace [61]. It encompasses three key dimensions: vigor—characterized by high energy, persistence, and resilience in overcoming challenges; dedication—a strong sense of purpose, enthusiasm, and personal significance in one’s work; and absorption—deep concentration and full immersion in tasks. Employees exhibiting high levels of engagement consistently demonstrate enthusiasm, perform their duties efficiently, and deliver high‐quality outcomes [62], while those who actively engage in job crafting tend to show even higher levels of work engagement [63]. Nurses with strong work engagement demonstrate passion and effectiveness in their roles, take a proactive approach to delivering quality patient care, consistently achieve superior performance outcomes [64], and typically exhibit greater energy along with more positive emotions in the workplace [65]. Such positive emotional states enhance their ability to focus on patients’ strengths and potential rather than their difficulties or limitations. This strengths‐based perspective fosters a more optimistic view of patient recovery and encourages care practices oriented toward patients’ capabilities. Consequently, this may enhance the quality of mental health care by providing improved discharge support and more effective assistance in helping patients attain their preferred community living arrangements [64]. Work engagement may serve as a key psychological motivator for implementing strength‐based care across various nursing specialties, including psychiatric nursing—a field where nurses often face challenges in maintaining positive emotions due to difficult experiences, such as patient‐related violence [24]. In the highly regulated setting of operating rooms, nurses’ responsibilities are more standardized and formalized, resulting in limited job autonomy. Providing regular education and training programs on job crafting for operating room nurses could help mitigate this limitation. Enhancing job autonomy fosters intrinsic motivation, as individuals perceive greater control over their work performance [44]. Work engagement may, at least in part, reflect a reversed causal relationship with collaborative job crafting, as conceptual work suggests that employees with higher levels of engagement are more inclined to proactively enhance their job resources, that is, to engage in job crafting [23]. Not all findings align completely with the JD‐R assumptions. For instance, Iida et al.’s longitudinal study did not reveal a significant long‐term correlation between team‐level job crafting and individual work engagement [23]. This suggests that job crafting’s effectiveness may depend on contextual and organizational factors, such as leadership support, team dynamics, and workplace culture. Moreover, these individuals are generally more motivated to engage actively in organizational initiatives [23]. Intervention strategies, such as job crafting diaries, can significantly enhance nurses’ self‐efficacy and encourage proactive job crafting behaviors. Equally important is fostering their involvement in decision‐making processes, as strengthening their voice and sense of participation is essential for promoting higher levels of work engagement [40].

Recent literature increasingly highlights the relationship between job crafting and job burnout among nurses [33, 34, 40]. When nurses face mild to moderate levels of burnout, the condition often goes underrecognized by both nurses and their managers, which can contribute to serious consequences for nurses’ physical and mental health, job performance, and commitment to the organization [51, 66]. África Martos Martínez and del [67] report that job crafting explains between 15.7% and 19.7% of the variance in burnout across four dimensions: personal impact, job dissatisfaction, motivational withdrawal, and social climate, among male nurses [67]. A high level of job crafting enables individuals to reshape how they perceive their work, build stronger workplace relationships, and modify the nature of their tasks, factors that can help reduce burnout while enhancing job satisfaction and overall well‐being [68]. Numerous studies have reported an association between job crafting behaviors and higher levels of work engagement and job satisfaction, as well as lower levels of burnout [2, 24, 28, 33, 34]. These findings suggest that nurses who actively modify their tasks, relationships, or perceptions of work are better able to utilize job resources and cope with challenging clinical situations. Given the modest yet meaningful impact of job crafting on nurse burnout, it is essential for both nurse managers and nurses to actively promote job crafting behaviors in clinical practice through targeted interventions, such as workshops and structured exercises [69].

Work shaping helps maintain a proactive balance between job demands and resources, fosters self‐directed work behaviors, and ultimately reduces the risk of job burnout in this group of healthcare professionals [70]. When nurses effectively manage their resources alongside their job duties, their productivity rises, leading to greater work commitment [50]. Employees with extensive work experience tend to experience positive emotions, such as well‐being, joy, and enthusiasm, along with improved mental and physical health [71]. Older employees often exhibit broader workplace networks and stronger interpersonal relationships, which may significantly contribute to higher levels of job embeddedness [72]. Job crafting has been shown to play a significant role in strengthening organizational commitment [50], as research indicates that employees who actively engage in job crafting behaviors tend to demonstrate greater loyalty and dedication, which in turn contributes to improved job performance [27]. Strong commitment not only enhances resilience to workplace stressors but also encourages proactive role shaping. Additionally, it has been shown that organizational commitment is positively associated with job performance [65], and that the inclusion of job crafting further amplifies this relationship. Encouraging job crafting among employees may therefore reduce turnover intentions while boosting engagement and overall job satisfaction. Moreover, the moderating role of affective commitment has been highlighted, suggesting that individuals with strong emotional ties to their organization may experience positive outcomes regardless of their level of job crafting [27, 73, 74]. The potential to enhance job satisfaction is notably increased when employees engage in job crafting alongside supportive factors, such as job security, adequate compensation, engaging work tasks, and positive relationships with immediate supervisors [35]. Nurses facing elevated levels of work stress often struggle to provide high‐quality care, which negatively affects their physical and mental well‐being, ultimately reducing job satisfaction [75]. Given their critical role in ensuring the safety of both donors and recipients, gaining a clear understanding of the link between job stress and job satisfaction is essential for maintaining high standards of nursing care [45].

Employee well‐being is observed when their basic needs are met through job crafting, which involves aligning work responsibilities with their values or needs to achieve better performance, satisfaction, and enjoyment at work [2, 76]. Previous studies demonstrate a positive impact of workplace design on nurses’ well‐being [2, 49]. The relationship between workplace design and the well‐being of healthcare system employees underscores the role of an effectively structured environment in promoting both well‐being and productivity [15].

The JD‐R assumes that the level of job resources (e.g., autonomy, social support, and job crafting) influences engagement and the risk of burnout. Analysis of the research indicates that active job crafting can enhance nurses’ motivation and job satisfaction [2, 23, 25]. However, results showing weaker or insignificant correlations suggest that job crafting may be less effective when faced with high job demands or insufficient organizational resources, which is consistent with JD‐R assumptions [23, 24]. In light of the JD‐R theory, job design can be interpreted as a moderating or reinforcing factor that mitigates the negative impact of high demands on burnout while increasing engagement, provided that sufficient resources are available. Adopting this theoretical approach confirms the usefulness of the JD‐R model for analyzing job crafting in nursing. For example, it could help to determine which dimensions of job crafting best reduce stress and support engagement in different professional contexts [23, 25, 28].

Many studies focus on the individual aspects of job crafting; there is growing evidence that its effectiveness is largely determined by systemic and organizational factors. For instance, leadership style plays a vital role: Leaders who encourage autonomy and professional development among their employees tend to foster greater engagement in modifying tasks and work processes [23]. Organizational culture also plays an important role. Organizations with an open culture that encourages innovation and cooperation are more likely to support job crafting initiatives. In contrast, rigid, hierarchical, or communication‐restricted environments can hinder nurses’ ability to actively shape their work [2, 28]. Research shows that limited staffing levels, high patient loads, and inadequate technological support can prevent nurses from implementing changes to their work, even when they are willing to take on a more active role [24, 57]. Taking these systemic factors into account improves our understanding of the fact that job crafting is not just an individual activity, but a process embedded in a broader organizational context. Future research could analyze how different management strategies and systemic interventions can either support or hinder effective job crafting.

Although numerous studies have highlighted the positive impact of workplace design on nurses’ engagement, satisfaction, and well‐being [2, 23, 57], it is important to recognize that this process comes with its own set of challenges. For example, overemphasizing individual job modifications can lead to role conflicts or misunderstandings within the team when the changes made by one employee are inconsistent with organizational expectations or the tasks of their colleagues [24, 25]. Furthermore, some studies have indicated that excessive job crafting, particularly in environments with limited resources, can result in cognitive or emotional overload [28]. This can undermine team effectiveness and limit the achievement of organizational goals, despite having a positive impact on the individual. Considering these potential limitations highlights the need for a balanced approach to job crafting, combining individual initiative with a clear organizational framework and team support. Future research could examine ways in which organizations can maximize the benefits of job crafting while minimizing the risk of conflict and overload [23, 25].

The dominant research design in the analyzed studies was cross‐sectional. This allows for the identification of relationships and correlations between variables, but not for conclusions about cause and effect. Hwang and Shin’s [37] indicate significant correlations between job crafting and person‐centered care or satisfaction [37]. However, they do not enable us to determine whether job crafting actually improves these outcomes, or if people who are more engaged are simply more likely to engage in job crafting [37]. Similarly, the cross‐sectional nature of studies examining the pathways between job design, engagement, and well‐being in nursing [2, 25] makes it impossible to determine the direction of these relationships. Unlike cross‐sectional studies, the longitudinal design employed in Iida et al.’s [23] study—a prospective cohort study of Japanese nurses—enables a partial evaluation of changes over time and observation of the translation of team‐level job crafting into engagement and other occupational outcomes in subsequent months [23].

Although most studies indicate positive correlations between job crafting and engagement or satisfaction, there are exceptions that demonstrate weaker or more variable relationships. For instance, a multisample study of nurses found that not all aspects of job crafting (e.g., cognitive and relational crafting) had a strong predictive effect on job performance. This suggests that the impact of different forms of job crafting may vary depending on the context. A prospective study showed that, when measured after 3 and 6 months, job crafting at the team level was not statistically associated with individual engagement [23]. Differences in results may be due to geographical and cultural contexts. For example, studies covering public and private hospitals in Spain have shown different relationships between job crafting and outcomes due to varying organizational conditions [25]. Meanwhile, research in Japan suggests that teamwork specifics and cultural norms may impact the relationship between job crafting and engagement in different ways [23]. Job crafting is influenced by cultural and regional differences. This can lead to variable effects in different healthcare contexts. Despite a large sample size (*N* = 2478), Iida et al. [23] found no association between team‐level job crafting and outcomes [23]. Studies with smaller samples (e.g., *n* = 207) have shown limited statistical power and potentially low detectability of effects [2]. The strength and statistical significance of the results may be affected by different sample sizes and measurement tools (e.g., different versions of the Job Crafting Scale). Not all studies confirm the same effects of job crafting. The strength and direction of these effects depend on the methodology used (cross‐sectional vs. longitudinal studies), the size and nature of the sample, the regional and organizational context, and the various forms and dimensions of job crafting. Therefore, the positive effects of job crafting are not universal, and their interpretation should consider the theoretical and empirical context of these differences.

## 5. Limitations

Although the conducted literature review offers valuable insights into the topic, it is subject to certain limitations concerning the scope of selected studies, the availability of recent data, and potential publication bias, which may impact the comprehensiveness and generalizability of the findings. Prominent among them are as follows: (I) Most of the research in this field has been conducted in Asia and has focused on Asian health systems. Despite the valuable insights gained from these healthcare systems, further research is warranted in other regions, particularly focusing on countries within the European Union and the United States; (II) the prepared paper does not provide a distinct analysis considering nurses’ specialties or specific work environments, such as primary care facilities, hospitals, or sanatoriums. In their study, Alhartni and colleagues showed that a higher job crafting score was associated with working in psychiatric hospitals and long‐term care units, working in outpatient departments, having a fixed work schedule, and working 6 to 8 h a day [46]; (III) although the results of the presented studies appear consistent, it is crucial to take into account the varying working conditions of nurses across different countries. The analysis does not consider country‐specific factors that could have implications at the local level; (IV) although a systematic approach was employed in the preparation of the review, certain relevant materials may have been omitted due to access limitations, such as the works of Yepes‐Baldó et al. and Baghdadi et al. [49, 50]. The limitation of this review relates to a significant proportion of the studies that were cross‐sectional. It makes it difficult to draw conclusions about the causal relationship between job crafting and professional outcomes, such as engagement or burnout. Additionally, regional and organizational differences may have influenced the results, limiting their generalizability. Limiting the review to materials published exclusively in English may have resulted in the exclusion of additional relevant studies.

Several directions for future research emerge from the limitations identified in this review. Firstly, as the majority of studies on job crafting among nurses have been conducted in Asian countries, future research should examine this phenomenon within other healthcare systems, particularly in European Union countries and the United States. Secondly, further studies should examine job crafting in different nursing specialties and work environments. For example, research comparing nurses working in hospitals, primary care settings, long‐term care facilities, psychiatric institutions, and outpatient departments could help to determine whether the relationship between job crafting and professional outcomes, such as work engagement, job satisfaction, and burnout, varies depending on the clinical context. Thirdly, future research should consider the organizational and contextual factors that may influence job crafting behaviors. Variables, such as leadership style, staffing levels, workload, autonomy, and organizational support, should particularly be analyzed as potential moderators or mediators of the relationship between job crafting and professional outcomes. Finally, this field requires methodological improvements. Many existing studies are based on cross‐sectional designs and self‐reported data, which limits the ability to infer causal relationships. Longitudinal studies and intervention‐based research could provide a deeper understanding of how job crafting develops over time, as well as whether organizational interventions aimed at supporting it can improve nurses’ engagement, well‐being, and retention.

## 6. Implications for Professional Practice

The findings highlight the pivotal role of job crafting within the health sector as a key strategy for advancing nursing practice on a global scale. The study emphasizes that work environment factors—such as job engagement, satisfaction, and burnout—are not isolated variables but are intricately connected to systemic organizational issues, including resource allocation, leadership practices, and institutional support structures. Given the increasing demands on healthcare systems worldwide, and the intensified resource and economic constraints, these findings carry profound implications for future practice (Table 3): (I) Health institutions should adopt a more strategic approach to workforce planning that ensures proper allocation of human and technical resources while preventing understaffing—a major contributor to nurse burnout. To complement strategic workforce planning, institutions should implement predictive staffing models grounded in patient acuity and workload metrics, thereby optimizing care delivery while minimizing staff burden. (II) Transformation of organizational culture. Empowering nurses to cocreate their work settings fosters autonomy, improves morale, and contributes to a stronger sense of professional identity and purpose. Nurse managers should be trained in participatory leadership approaches that encourage bottom‐up feedback and decision‐making. (III) Mental health and well‐being programs. The connection between personal well‐being and job performance underscores the need for holistic well‐being programs, such as mental health check‐ins, resilience training, peer support, and stress‐relief activities. (IV) Institutional support frameworks. As Ujoatuonu et al. [77] emphasize, administrative support, through reduced overtime and access to leisure programs, can significantly enhance job satisfaction [77]. This necessitates that leadership extend its role beyond mere performance monitoring to actively fostering the holistic well‐being of staff. Practical steps may include flexible scheduling, recognition programs, and accessible career development pathways. (V) The factors facilitating or hindering performance differ considerably across various types of nursing units. Institutions should regularly assess these contextual differences and adapt interventions accordingly. Tailored training programs, mentorship structures, and performance feedback systems that are unit‐specific will likely yield better engagement and retention outcomes. (VI) Leadership capacity building in environmental design. Nurses who take the initiative in job crafting tend to display more positive attitudes and improved psychological well‐being [2]. Future practice should invest in developing such leadership potential among nurses through structured programs that focus on change management, systems thinking, and interprofessional collaboration. (VII) Data‐driven management and evaluation. Ongoing research into job crafting should be incorporated into continuous quality improvement efforts. Institutions should collect and analyze data on nurse satisfaction, burnout rates, and turnover as part of standard operational dashboards.

**TABLE 3:** Implications for nursing management practice.

Feature	Managerial action	Increased benefit	Priority
Workload	Implement predictive staffing models	Reduced burnout	High
Leadership	Implement participatory leadership approaches and encourage bottom‐up feedback	Increased engagement	High
Psychological well‐being	Mentoring, peer support, resilience training, and mental health programs	Enhanced well‐being	Medium
Training programs	Development of job crafting support programs	Enhanced job satisfaction	High
Monitoring	Development of unit‐specific performance feedback systems	Increased engagement	High
Job crafting potential	Leadership capacity building in environmental design.	Improved psychological well‐being and professional autonomy	Medium
Evaluation	Implement data‐driven management through regular monitoring of nurse satisfaction, burnout, and turnover indicators	Continuous quality improvement and improved staff retention	High

In summary, the findings advocate for a transition from reactive, task‐focused management of nurses to a proactive, person‐centered approach that recognizes nurses as essential stakeholders in the design and development of their work environments. Institutions that integrate these implications into their strategic frameworks will be better positioned to maintain a resilient, motivated, and high‐performing nursing workforce.

## 7. Conclusions

The findings of this review clearly demonstrate a strong association between nurses’ active engagement in job crafting and their intention to remain in their respective positions. This issue is especially critical given the increasing rates of nursing turnover, which jeopardize both the quality of clinical care and the stability of healthcare systems. High nursing turnover generates serious organizational and emotional consequences: It leads to excessive strain on the remaining staff, exacerbates professional stress, and contributes to burnout, which in turn reduces their job satisfaction and the quality of care provided. For patients, this means a lower level of safety, more frequent interruptions in the continuity of care, a higher risk of medical errors, hospital‐acquired infections, and even increased mortality rates. For this reason, identifying and strengthening elements of the work environment that foster long‐term professional commitment among nurses should become a priority for policymakers and healthcare executives. Particularly significant are initiatives that promote enhanced professional autonomy, equitable allocation of responsibilities, access to training, and career development opportunities, alongside mechanisms supporting employees’ psychological well‐being. Our review confirms the necessity of implementing comprehensive human resource management strategies in nursing, grounded in staff dialog and systematic evaluation of working conditions. Reducing turnover, enhancing care quality, and strengthening the healthcare system are achievable only through the deliberate, evidence‐based shaping of the work environment. Furthermore, a limited number of studies meeting the criteria of this literature review have examined the relationship between work environment design, highlighting a critical area for future research in this field.

## Funding

This work was supported by Polish Ministry of Science and Higher Education, grant no. SUPB.RN.21.234.

## Conflicts of Interest

The authors declare no conflicts of interest.

## Data Availability

Data sharing is not applicable to this article as no datasets were generated or analyzed during the current study.
